# Unconscious knowledge: A survey

**DOI:** 10.2478/v10053-008-0081-5

**Published:** 2011-03-04

**Authors:** Luís M. Augusto

**Affiliations:** Institute of Philosophy, Faculty of Letters, University of Porto, Portugal

**Keywords:** unconscious/implicit knowledge, unconscious mental processes (perception, learning, memory, thinking, decision making), measures of unconscious knowledge

## Abstract

The concept of unconscious knowledge is fundamental for an understanding of human thought processes and mentation in general; however, the psychological community at large is not familiar with it. This paper offers a survey of the main psychological research currently being carried out into cognitive processes, and examines pathways that can be integrated into a discipline of unconscious knowledge. It shows that the field has already a defined history and discusses some of the features that all kinds of unconscious knowledge seem to share at a deeper level. With the aim of promoting further research, we discuss the main challenges which the postulation of unconscious cognition faces within the psychological community.

##  

“Knowing,” in short, may, for aught we can see beforehand to the contrary, be *only one way of getting into fruitful relations with reality*…. (James, 1904)

## Introduction

Contemporary psychology speaks of *unconscious knowledge* (also *unconscious cognition, implicit knowledge/cognition, tacit knowledge*) to refer to cases in which subjects display available knowledge to which they lack conscious access. While this is not controversy-free in psychology, a significant part of the psychological community attributes to this claim a scientific status, contrary to what happens in the case of the psychoanalytical postulation of an unconscious mind. Part of this attribution of scientific status by a community that is not remarkable for being generous with this acknowledgement is due to the methodological approaches used by the diverse psychological disciplines: They all follow the strict rules of the scientific method.

Although the concept is still largely unknown outside the field of psychology, scientific hypotheses on unconscious knowledge have proven to be (and promise to be) extremely important in many fields involving the processing of knowledge. Education, medical care, knowledge management, and consumer behaviour are examples of a few fields that already benefit (or will potentially do so) from the findings obtained from research into this particular subject matter. Despite the research being carried out in many psychological disciplines, the vast majority of the psychological community seems to have little or no knowledge of the subject as a whole. This is evidenced by the scanty or even absent referencing across the many fields that deal with the topic, as a glimpse of much of the recent work cited in this paper – with the exception of studies in implicit learning, implicit memory, priming, and anaesthesia, which display a fair amount of cross-referencing – will show. While it seems this is now slowly changing, with studies extending into other fields (see e.g., the study of Van der Kamp, Oudejans, & Savelsbergh, 2003, which puts into relation the dichotomy between implicit and explicit learning and the distinction vision for action vs. vision for perception), still there is no unified discipline of unconscious knowledge. This paper aims to provide a unified view of this discipline.

This paper does not approach the Freudian dynamic unconscious extensively. The main reason for this is the lack of a consensus concerning its scientific status; this is certainly open to discussion, but this will not be undertaken here. However, a brief treatment of Freud’s influence in the field of studies on unconscious knowledge is mandatory. This survey does not include all the cases in which one can speak of unconscious knowledge; here, we discuss only those based on robust neurophysiological and/or behavioural observational evidence.

## Unconscious Knowledge: What, How Distinct, and Why?

### What?

In psycho-cognitive terms, *knowledge* can be defined as information or data about the environment (roughly: sensory input) that can be acquired, stored, and retrieved by living organisms with a more or less complex nervous system, with a view to securing their wellbeing. *Cognition* can be defined as the actual process of acquisition, storage, and retrieval of knowledge; cognition is therefore the skill of dealing with knowledge (Neisser, 1976). The two, knowledge and cognition, are thus easily confounded, the two terms being harmlessly – if not entirely correctly – interchanged in the field of cognitive psychology. In the case of humans, the information or data comprising knowledge can, in principle, be more or less expressible through verbal language, without the subject being necessarily or actually capable of doing so; infants, for instance, cannot express knowledge verbally, but we do not say that they are not cognitively active. Moreover, subjects may be completely unaware of a particular cognitive operation that is being carried out within them, and they may be unable to infer indirectly that such an activity is taking place because of a lack of overt behaviours. Thus, besides relying on direct verbal reports, we can assume the occurrence of a cognitive process based on a plethora of behaviours, overt or covert, that we believe indicate that a subject is acquiring, storing, or retrieving information. In other words, there are *internal mental processes* (representing, believing, learning, memorizing, etc.) taking place. In general, cognitive psychology reposes on these assumptions (cf. Neisser, 1967, 1976).

In the cases where subjects exhibit behaviours that indicate that they possess knowledge but seem both unaware of that possession and unable to verbalize it, we assume that they have unconscious, or implicit knowledge. More specifically, availability of knowledge in the absence of conscious accessibility is what mostly distinguishes *unconscious* from *conscious* (also *explicit*) *knowledge*. *Unconscious knowledge* refers to knowledge that is revealed by task performance alone, subjects being unaware that they are accessing it, whereas we speak of conscious knowledge when subjects are aware of possessing and accessing it (Schacter, 1992). A useful way of characterizing this epistemic availability in the face of conscious inaccessibility is by appealing to *metaknowledge* (e.g., Dienes & Perner, 2002): One can speak of unconscious knowledge when subjects lack metaknowledge concerning their own positive epistemic states, that is, states in which they possess knowledge. In other words, subjects cannot form a higher order representation about a lower order one. For instance, a subject with blindsight (see below) who, when forced to guess, correctly identifies a cross on a screen, has a lower order representation that there is a cross on the screen; however, this subject is incapable of representing this information to themselves with a higher order representation. That is, the subject cannot say, “I see a cross on the screen”; seeing the cross on the screen is not a conscious thought in this case (e.g., Rosenthal, 2005, p. 185). Returning to the availability-accessibility distinction, we can say that while the sight of a cross on a screen is available to the subject with blindsight, it is not consciously accessible to them.

The claim is often stronger than this: Unconscious knowledge is not just knowledge that fails to reach consciousness or a higher order conscious thought. Unconscious knowledge is claimed to be qualitatively different from conscious knowledge and acquired by means or cognitive pathways distinct from those that produce conscious knowledge (e.g., Greenwald, 1992; Reber, 1989, 1992a, 1992b; Schacter, 1992). Accordingly, we use experimental methods that can appropriately probe unconscious knowledge: Subjects are presented with stimuli that they cannot bring explicitly to consciousness but we can, nevertheless, show that they have cognitively processed those stimuli – that is, there has been unconscious perception, unconscious storage, and unconscious retrieval. In these experimental methods, subjects are unaware of the stimuli, because they are too weak, brief, complex, or are masked, etc. Other reasons are if the subjects are in a state of complete unconsciousness (sleep, coma, anaesthesia, etc.), if they cannot be conscious of certain kinds of stimuli (clinical conditions, such as blindsight, hemineglect, prosopagnosia, etc.), or even because their attention has been diverted to another demanding task.

If it is true that, in principle, *unconscious cognition* refers to cognitive processing which takes place completely outside consciousness (information is learned, stored, and recalled in an unconscious way, as in non-associative, associative, and motor forms of learning, for instance), it is nevertheless important to realize that knowledge acquired and stored in this way can be consciously retrieved (e.g., operant conditioning). It is also important to recognize that a knowledge base which can be consciously accessible in principle (*explicit memory*) can be probed unconsciously (e.g., subliminal perception; see Sweatt, 2003, p. 7, for a diagram capturing these distinctions). Although these distinctions should be kept in mind, we believe we are dealing with unconscious cognition: Again, what allows us to talk of unconscious knowledge is the fact that the subjects lack metaknowledge, in the sense that they are unable to specify how they acquired, or that they are accessing, portions of their knowledge bases.

Epistemologically, to speak of unconscious knowledge is to say that unconscious mental processes (e.g., beliefs, thoughts, etc.) yield knowledge, which makes the expression simultaneously superfluous and erroneous: Whether yielded by conscious or by unconscious mental processes, knowledge is, of course, knowledge simpliciter. Nevertheless, given that it seems that unconscious knowledge is to a great extent qualitatively different, we see no harm in using this expression, at least in situations in which one wishes to make clear that the knowledge one speaks of is processed by means of wholly or largely unconscious information processing.

This ranges from basic perceptual processing to spontaneous problem solving, and the kinds of stimuli that prompt such processes range from low-intensity, brief, or masked physical stimuli to highly complex systems of rules (linguistic, social, cultural, etc.). Although verbal reports by subjects are much used in experimentation, one often has to focus on non-verbal behavioural responses, whether overt or covert; this is especially important when approaching unconscious processes in situations involving clinical conditions, such as left visuo-spatial neglect or blindsight, in which subjects report absence of awareness of stimuli and therefore claim not to hold any beliefs or thoughts regarding them.

### How distinct?

Just how qualitatively different unconscious knowledge seems to be can be summarized as follows:

1. The feature that fundamentally distinguishes unconscious from conscious knowledge is the fact that the former appears to be purely *procedural*, while the latter seems *declarative* in nature. By *procedural*, we mean that this kind of knowledge is expressed in procedures or performance alone, not being, in principle, verbalizable; in other words, subjects exhibit a dissociation between performance and reportability, being incapable of verbally expressing actions they perform and behaviour they display.^1^ Common examples of this kind of knowledge are riding a bike, speaking a language as a native speaker, judging faces, etc. However, the classification of unconscious knowledge can better be applied to other instances of behaviour without awareness, such as that displayed in the case of certain perception and cognition disorders in which it is hypothesized that subjects are in possession of specific knowledge while incapable of accessing it for neurological reasons (lesion, malformation, etc.). The procedural versus declarative distinction is also common in the field of research of memory due to the obvious connections between memory and knowledge (namely knowledge as a data base, e.g., Cohen & Squire, 1980; Squire, 1982, 1986), though further, higher distinctions commonly apply in the case of memory (see Figure 3).

2. It appears that knowledge acquired and stored in an unconscious way is, when durable,^2^ more robust than that acquired in an explicit mode; a conclusion drawn from the finding that many unconscious kinds of knowledge are not lost in amnesia (e.g., Graf, Squire, & Mandler, 1984). This feature appeals to Jackson’s principle, according to which the degree of resistance of a mental function is directly related to its antiquity in a species; it is hypothesized that this robustness is accounted for by virtue of the precedence, in evolutionary terms, of unconscious learning modes (Reber, 1989, p. 232; 1992b, p. 109).

3. This kind of knowledge appears to be *holistic* (vs. analytic) in that the knowledge representations are solely atomic, failing to distinguish the different components: For example, the representation of a rule or compositional structure such as *P* & *Q* is not decomposed in its constituents *P* and *Q*; it has no internal structure (Fodor & Pylyshyn, 1988; Roberts & MacLeod, 1995). Given that this feature is somehow connected to linguistic aspects, this might better explain the procedural nature of unconscious, non-verbalizable knowledge as compared to many other concurrent theories (see Roberts & MacLeod, 1995, p. 300).

4. *Routinized* and *inflexible* seem also to be distinctive features of unconscious knowledge, with performance collapsing when alterations are introduced in experimentally controlled tasks (e.g., Bayley, Frascino, & Squire, 2005). This might well be a reflection of the fact that this kind of knowledge appears to be tied to surface characteristics (visual, auditory, etc.). For example, Berry and Broadbent (1988) found that if certain tasks (transport or person interaction tasks) were perceptually similar, transfer of performance was actually verified; Reber (1969) showed a memory advantage when changing the letters in a grammar task while keeping the same grammar; Mathews and colleagues (1989) also reported good transfer with only a change of letters. However, “despite the surprising ability of subjects to transfer across domains,” as Dienes and Berry (1997) summarize it, “the knowledge is partly perceptually bound and transfer is not normally complete even when a simple mapping is known” (p. 8). Most importantly, this feature can also be accounted for by lack of conscious access in that knowledge bases which are accessible in a conscious way are subject to corruption due to a multitude of factors, as has been long known (see Bartlett, 1932, and Loftus & Palmer, 1974, for two classical examples). 

5. Predictably, it is claimed that unconscious knowledge is *independent* of explicit knowledge (e.g., Willingham & Nissen, 1989). Tulving (Tulving, 1985; Hayman & Tulving, 1989), in the context of research on memory, spoke of “stochastic independence” to express the probability that success in one measure is independent of whether there is success or failure in the other measure, the measures being of implicit and explicit memory. In the domain of unconscious learning, this independence was also verified; for instance, Berry and Broadbent (1984, 1987, 1988) reported that improvement of practice in performance is not accompanied by a similar improvement in verbalization. Again, this feature is in agreement with the overall procedural (vs. declarative) character of unconscious knowledge.

6. Other, more recent predictions, which have been tested only insufficiently or not at all, are that unconscious knowledge is age- and IQ-independent, and that it should show lower population variance (see Reber, 1992b). Despite the lack of robust experimental results, the above characteristics appear to support these predictions.

One important aspect to bear in mind is that these features were unveiled chiefly by research on very specific domains of unconscious cognition, dealing mainly with artificial grammar learning, control of complex systems, and sequence learning; that is, they are associated with implicit learning (see Artificial Grammars and Simulated Systems section). However, data obtained from research on unconscious processes not immediately aiming at establishing cognitive features fits well into these findings, thus confirming the belief that all kinds of unconscious knowledge are essentially similar, at least at the deepest level (Reber, 1989, p. 219). More than a skill (that is, at a deeper level), procedural knowledge is a set of procedures, instructions, even algorithms, or just structures or patterns that are implementable rather than describable. Subjects act in a goal-directed and often skilled way without being aware that they do so, and, when probed, without being able to say what it is they draw on. This is commonly the case in habituation, in which irrelevant stimuli are increasingly ignored, but it can also be elicited by priming. For example, Lewicki and colleagues (Lewicki, Hoffman, & Czyzewska, 1987) primed subjects to locate a target following a complex and non-salient pattern, and Neumann (2000) led subjects to feel either guilt or anger by priming their attribution of emotions, something we in principle do without being aware of relying on rules from our knowledge bases. Conditioning, classical or operant, is perhaps an even better way to elicit procedural knowledge. Here, the subject is basically unaware of the associations and responses established: In fact, we can, in a way, say that subjects unconsciously *know* the procedures, instructions, or rules (“if conditioned stimulus *X* is present, then produce conditioned response *Y*”; “if environmental cue *S* is present, then do *R*”) even when they are not aware of the whole situation. A good illustration of this is evaluative conditioning, or the conditioning of affective responses, which does not require awareness of the contingencies and often results in unconscious activation of goal pursuits (e.g., Custers & Aarts, 2005). Furthermore, this can in turn be connected with the holism vs. analyticity feature discussed. In the examples above, the subjects are not aware of the associations (representable as *P* & *Q*) and response activations (*P* → *Q*). The subject is incapable of decomposing them into their constituents; namely, many results within both the complex systems paradigm (see Artificial Grammars and Simulated Systems section) and the somatic marker hypothesis (see The Somatic Marker Hypothesis section), in which subjects are confronted with situations of the kind “if *P* is the case, then do/expect/don’t do/... *Q*”, can be accounted for by this feature. Regarding habituation, we can hypothesize that something similar takes place, with the subject being unconsciously instructed to ignore the presence of a stimulus (“if stimulus S is present, do *R* [= ignore *S*]”). 

There is, nonetheless, one feature claimed by research on unconscious cognition that does not seem to be applicable to all situations of unconscious knowledge, and that is *abstractness* (see Reber, 1969, 1989). In fact, if such a feature seems to apply to the learning of rules, namely of the complex kind, it is not so in the case of mental representations in other situations in which subjects display unconscious knowledge. For this reason, this feature has been omitted from the list above. Moreover, in the case of rule learning, as seen, the holistic, indecomposable character of unconscious representations seems to explain better the reason why subjects cannot verbalize their knowledge.

### Why?

The postulation of a specifically unconscious kind of knowledge makes sense for many reasons. Firstly, it is quite clear that we are not aware of all percepts being simultaneously processed by our perceptive and cognitive apparatus; at best, we are only conscious of one or a few percepts at a time. Nevertheless, we do not cease acting; we continue to respond to the environment in ways that show that we are knowledgeable of it. This is particularly so in the case of automatized actions, such as driving a vehicle or typing – situations in which one is not at all conscious of these specific actions and yet carries them out with the necessary expertise. To invoke these situations is, however, often a source of much criticism, which challenges theories of unconscious knowledge because they can be easily brought to consciousness, though they are difficult or even impossible to verbalize. This criticism is countered with other examples, such as speaking a mother language: Most native speakers of a language are incapable of saying how they speak the language and what rules they follow. However, they speak it correctly^3^ and fluently, and are very good at spotting mistakes. Given the early age of the learners and the absence of a formal strategy of learning, it is only plausible that this system of grammatical rules is learned unconsciously.

It also makes sense, from the evolutionary point of view, that if consciousness is related to later developments in the human species – as it likely is, because apparently only animals possessing the neocortex^4^ (the mammalians) seem to be capable of (self-) consciousness (e.g., Eccles, 1992), then an unconscious form of perceiving and learning must have preceded the first steps of human evolution. The hypothesis of a dual visual stream, discussed in detail below, supports this evolutionary view. Humans with lesions in the conscious visual stream, the ventral stream, have to operate on a basis of data processed in an unconscious way by the dorsal stream, earlier in evolutionary terms (e.g., Milner, 1997).

This equates with postulating that animals, like reptiles and fish, which do not have a neocortex or a homologous structure, also have knowledge, albeit only of the unconscious kind. This is only in accord with one of the principles of contemporary evolutionary theory, the principle of commonality, stating that evolutionary earlier functions and forms are present across species (see Reber, 1992b, pp. 112, 120). Besides the evolutionary meaning, this is another good reason for referring to unconscious knowledge, given that we might feel reluctant to attribute conscious knowledge to other animals, yet they appear to process information in a very successful way.

Finally, we have many reasons to believe that humans begin to construct their knowledge bases, if not in a pre-natal state, immediately post-birth and throughout early infancy. This is a stage of development in which mental life is thought to be, for the most part, unconscious (e.g., babies sleep most of the time; verbal language, apparently intimately connected with consciousness – or some degrees/kinds of it, is mostly absent in early infancy, etc.; for studies in cognition involving pre-natal and early infancy development, see e.g., Fifer et al., 2010; Kisilevsky, Hains, Jacquet, Granier-Deferre, & Lecanuet, 2004; Tarullo, Balsam, & Fifer, 2010).

## History and Current Theories and Trends

### History

#### Freud and the Unconscious

Although the conception of an unconscious or, simply, of unconscious mental processes, emerged long before Freud (e.g., Ellenberger, 1970), contemporary research on unconscious knowledge is inevitably connected to the Freudian unconscious, and it is thus essential to address this connection. To begin with, it is a connection that many contemporary experimental psychologists vigorously reject, and one that not a few contemporary practitioners and sympathizers of psychoanalysis seek to strengthen. If the former group see the postulation of the Freudian unconscious as lacking in scientific status (as far as the dynamic, or largely irrational and chaotic unconscious is concerned), the latter see the experimental results as corroborating and further developing the psychoanalytic theories (e.g., Davou, 2002; Ekstrom, 2004).

The rejection of the connection by contemporary psychologists is typically a leftover from behaviourism, which until recently dictated matters and methods in psychology. This is so much so that more often than not the term *unconscious* is altogether dropped in favour of the less charged *implicit*, or tacit (e.g., Schacter, 1992). However, the radical view that the dynamic unconscious is an altogether dispensable postulation is probably more often and more vigorously advocated from outside psychology proper (e.g., O’Brien & Jureidini, 2002). In this field, it has frequently been acknowledged that the dynamic unconscious is not irrelevant to experimental psychology; on the contrary, it provides it with important theoretical material. Shevrin and Dickman (1980), for example, claimed that the tripartite dynamic characterization of the unconscious – psychological, active, and different in character from conscious processes – has been incorporated in many experimental studies. Based on this notion of *unconscious mentation* and on experimental studies on selective attention, subliminal perception, and visual phenomena involving perceptual processing (such as retinal image stabilization and binocular rivalry), the authors conclude, against strong forms of behaviourism, that “behavior cannot be understood without taking consciousness into account and that conscious experience cannot be fully understood without taking unconscious psychological processes into account” (Shevrin & Dickman, 1980, p. 432). 

Freud did not invent the wheel and much of his merit lies in having been able to put together many intuitions that abounded at the time he started his research in neurology. In fact, his development of a psychology of the unconscious mirrors, in many ways, the “non-scientific” sources from which he directly or indirectly drew. For instance, one field in the 1800s in which unconscious (or somehow akin processes) of thought were being avidly researched was animal magnetism, and the methods applied were, among others, suggestion and hypnosis (e.g., de Faria, 1819/2005). The latter was precisely the first method used by Freud in his first wanderings into the realm of the unconscious, before developing more idiosyncratically dynamic techniques, such as dream analysis and free association. None of these was a scientifically recognized method of experimentation and research, but they were necessary to found a discipline that was above all an analysis of human psychical life with a view to therapeutic ends.

This is not the place to defend the scientific status of the dynamic unconscious, nor is the aim here to sanitize it, but it is important to note that Freud did not always write of it in terms that can be judged by many as non-scientific. As a matter of fact, only late in his development did he speak in terms perhaps too vivid for more conservative minds. Here, he discussed the *id*, a somehow structural rough reformulation of his earlier topographic concept of the unconscious that greatly emphasized the “compulsive” (*triebhaft*) character of unconscious psychic contents after a reformulation of his theory of “drives” or “instinctual needs” (*Triebe*; cf. Freud, 1920/1961, 1923/1961).^5^ Then, he analogically spoke of the id as “a chaos, a cauldron of seething excitations” (Freud, 1933/1964, p. 73) and, less vividly but perhaps still in an overly unorthodox manner, “a striving to bring about the satisfaction of the instinctual needs subject to the observance of the pleasure principle” (p. 73). He had said something similar in earlier writings in different terms (Freud, 1900/1958, 1915/1968), but it is in this later text that the wild and wholly irrational aspect of the unconscious is emphasized. This does not help Freud today:^6^ It is this aspect, together with other problematic issues (such as unconscious moral self-censorship and repression), that makes most opponents stick to their dismissal of the unconscious that Freud referred to as *dynamic*, that is, as bringing about an incessant state of psychic conflict between its irrational drives and the resistances of its conscious counterpart. However, in Freud (1940/1964), he offers a final development of a concept of the *unconscious* partly in terms that are manifestly not alien to those of some quarters of contemporary experimental psychology, as shall become evident below:

We know what is meant by ideas “occurring” to one – thoughts that suddenly come into consciousness without one’s being aware of the steps that led to them, though they, too, must have been psychical acts. It can even happen that one arrives in this way at the solution of some difficult intellectual problem which has previously for a time baffled one’s efforts. All the complicated processes of selection, rejection and decision which occupied the interval were withdrawn from consciousness. We shall not be putting forward any new theory in saying that they were unconscious and perhaps, too, remained so. (Freud, 1940/1964, pp. 283-284)

This is what Freud called the *descriptive unconscious*, or the psychic content that is latent and only temporarily outside the grasp of consciousness. Interestingly, this is a return to the more contained tone used by Freud in the formulation of the first topographical theory of the unconscious (Freud, 1915/1968). In addition, it suggests a symbiotic development of Freud’s concept of the unconscious and that of experimental psychology (see next section), more than perhaps a one-sided influence regarding any of the two parts.

#### Unconscious Cognition from Early Experimental Psychology to Cognitive Psychology

 Before the psychoanalytic concept of the unconscious was more clearly elaborated (Freud, 1900/1958), scientific psychology, still in its beginnings, already showed an interest in unconscious processes: Hypnotism and suggestion, somnambulism and automatisms (e.g., Charcot, 1882; Janet, 1889; Sidis, 1898), as well as unconscious sensations and perception (e.g., Binet, 1896; Fechner, 1860; Peirce & Jastrow, 1884), were some of the most investigated and experimentally researched mental phenomena in the later decades of the 19th century. However, the rise of behaviourism in American psychology soon banned these phenomena from the field of psychology, partially allowing studies only on “behaviour without awareness”.^7^ Eventually, this gave origin to a whole industry of experimentation on mainly *subliminal perception*, or *subception*, which had also started in the late 1800s.^8^ The basic hypothesis behind this research was – and still is – that we cannot equate discrimination with awareness, or, in other words, that much information is processed unconsciously, a conclusion reached already in the mid-1880s by Peirce and Jastrow (1884). In an experiment – in which they were the subjects – with extremely low differences in the application of the same kind of stimulus (pressure by means of weights), they verified that though they would claim not to have felt the difference (they showed zero confidence), yet still they got it right in approximately 60% of the cases, that is, above chance. Their main point was that, having zero confidence, a subject would be expected to get it right as many times as they got it wrong (chance results). As subjects “guessed” correctly in 60% of the cases, it showed that there was indeed unconscious perception of the difference of stimuli – which, in turn, questioned Fechner’s (1860) absolute and difference thresholds, unless his notion of *unconscious sensations* was made clearer in the context of a theory of subliminal perception.^9^

 Experimental studies on subliminal perception were fundamental for contemporary psychology in that, at a time when unconscious processes were by and large dismissed from serious psychological research, they provided abundant data and formulated important conclusions that helped to shape today’s approach to unconscious mentation. Soon after an important controversy regarding subliminal perception (Eriksen, 1956; Goldiamond, 1958; Lazarus, 1956; Lazarus & McCleary, 1951; for a review, see Dixon, 1971), Spence and Holland (1962) reported the paradoxical experimental result that awareness somehow restricts perception and cognition. In detail, they verified that (a) registration of stimuli is independent of awareness; (b) the effect of impoverished, that is, subliminal stimuli vary inversely with their intensity; (c) impoverished stimuli follow laws independent from those that rule conscious perception; (d) awareness of stimuli restricts their effect on recall of associated words. 

In face of these results, and in the wake of cognitive psychology, A. S. Reber (1967, 1969) opened up the path to research focusing explicitly on unconscious cognition; the now more cognitively directed assumption, was – and still is – that often we do not know that we know. More recently, with advances in neurophysiology, other unconscious processes were added to what is now a massive field of research, when the many diverse approaches are placed under the same banner. The next part of this paper describes the main results obtained in the different current approaches. The main selection criterion is the “knowledgeable” behaviour of subjects in contrast with their lack of metaknowledge (as defined above).

### Current theories and trends

#### Unconscious Perception

It is impossible to speak of perception without appealing to virtually all aspects of psychology, as it involves the complex phenomenology that begins with a stimulus and encompasses various levels (physical, cognitive, affective, etc.) and factors (attention, motivation, etc.) of information processing. To speak of *unconscious perception* is even more problematic, because it is implied that not only can subjects receive/discriminate a stimulus without awareness of that fact, but they can also process it in an unconscious way, in a kind of unconscious phenomenology. This goes against many robustly implanted and historically resistant philosophical and psychological assumptions (e.g., Brentano, 1874/1973; Descartes, 1644/1983; Locke, 1690/1959). However, as seen in the previous section, scientific psychology questioned strongly this idea from its very beginnings. In fact, Fechner’s (1860) still confused notion of *unconscious sensations* and, later, Peirce and Jastrow’s (1884) conclusions on small differences in sensation, aimed to show that we can, and more often than not do, discriminate stimuli from the environment in a wholly unconscious way. The data below, on *unconscious visual perception*, as gathered from clinical conditions, such as blindsight, prosopagnosia, and left visuo-spatial neglect, strongly supports this. The fact that in experiments in masked priming subjects can process meaning shows that unconscious perception can take place at higher levels of processing and, in turn, data from studies in anaesthesia and coma appear to corroborate the hypothesis that humans build and/or activate extensive parts of their knowledge bases in an entirely unconscious way.

##### Conscious versus unconscious visual pathways

**Figure 1.**
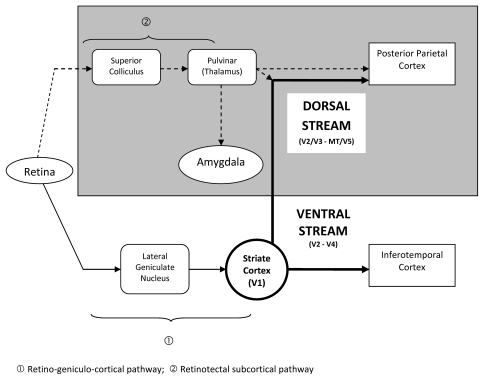
A model of unconscious (gray area) and conscious pathways of visual processing (not all possible connections are shown).

It will be interesting to start this survey on contemporary theories of unconscious knowledge with neurocognitive approaches postulating cerebral correlates of conscious and unconscious cognitive processing, namely regarding vision. One of the most productive is the hypothesis of a dual visual system of parallel, normally interacting, but greatly independent functionally differentiated cortical pathways, one providing what has been termed *vision for action*, and the other responsible for *vision for perception* (e.g., Milner & Goodale, 2007). Anatomically, and sketchily, both streams start in the striate or primary visual cortex (V1); the ventral stream projects to the inferior temporal cortex and the dorsal stream to the posterior parietal cortex. It is further hypothesized that there are subcortical visual pathways to the dorsal stream that bypass V1 (e.g., Berman & Wurtz, 2008; Striemer, Chapman, & Goodale, 2009), an important model to explain unconscious visual perception in the case of extensive damage or even absence of V1 (see Figure 1). In terms of function, it is thought that the dorsal stream is responsible for the use of information about objects (shape, size, orientation, motion, location) for guiding action, but not for their identification with a view to storage and recall in a knowledge base – the job of the ventral stream, thus justifying the often used labels of “how”/“where” and “what” pathways for the dorsal and ventral streams, respectively. Strong evidence for this anatomico-functional distinction comes from specific dissociations in what might be seen, in general terms, as an object versus action semantics dissociation (Hodges, Spatt, & Patterson, 1999); for instance, patients with visual agnosia displaying skilful mechanical action, and patients with optic ataxia showing normal object identification.

Of import for this survey is the fact that this anatomico-functional distinction corresponds to a segregation between conscious and unconscious processing of visual stimuli. In fact, given the different objectives or outputs of each stream (the evolutionarily earlier dorsal stream guiding action and the more recent ventral stream working for perception) means their processing takes place differently as far as consciousness is concerned. Thus, because action does not require high-frequency, fine-grained spatial representations of objects, but merely low-frequency metric data, it is claimed that the dorsal stream processes its visual input in a wholly unconscious way, whereas the ventral stream requires some degree of consciousness (e.g., Bridgeman, 1992; Goodale & Milner, 1992; Milner & Goodale, 2007). Anatomically and functionally, this dissociation may also imply two distinct visual pathways to the limbic system, making for the ventral and dorsal visuolimbic pathways, the latter being seen as implicated in the unconscious emotional processing of stimuli of relevance for the individual (cf. Bauer, 1984, p. 464). Whether or not this is the case (see Breen, Caine, & Coltheart, 2000, for a rejection and alternative model), the colliculus-pulvinar pathway to the amygdala, a pathway that also provides visual input to the dorsal stream, seems to account for emotional responses to visual stimuli in the case of damage or absence of V1 (e.g., Hamm et al., 2003; Morris, Öhman, & Dolan, 1999; see Figure 1).

 Within this framework of a dual visual system differentiated into conscious and unconscious pathways, the puzzling visual phenomena of blindsight, prosopagnosia, and left visuo-spatial neglect, which are all said to imply unconscious knowledge in the absence of conscious visual processing, appear to be attributable to the sparing of the dorsal in the damage or total destruction of the ventral stream (Milner, 1995) or, in the absence of striate cortex, to a subcortical colliculus-pulvinar visual pathway to the dorsal stream or to the amygdala (Johnson, 2005; Palermo & Rhodes, 2007). However, the reader should be aware that the model is not without challenges: For instance, it is argued that the need for the distinction between the two visual streams, ventral and dorsal, is not obvious or justified (e.g., Andersen, 2002; McFarland, 2002). In fact, functionally, the distinction is perhaps far from being as clear-cut as its supporters claim it to be. One can even go further to divide both or one of the streams, as Rizzolatti and Matelli (2003) do, which greatly complicates a model that seemed to be useful precisely because of its functional simplicity (the dorsal stream busies itself solely with guiding action, and the ventral stream works for perception alone). Against this criticism, recent studies continue to find evidence that seem to support the hypothesis of the anatomico-functional distinction (e.g., Almeida, Mahon, & Caramazza, 2010). 

###### Blindsight

*Blindsight* (e.g., Cowey, 2004; Weiskrantz, 1986; Weiskrantz, Warrington, Sanders, & Marshall, 1974) is the ability of human individuals with scotomata (blind regions of the visual field) caused by damage to V1 to somehow discriminate visual stimuli. More specifically, when “forced” to guess, they can distinguish shapes, such as O and X, they can discriminate line orientations, and are capable of differentiating gratings from homogeneous fields. These are feats that are indeed perplexing, given that the patients claim either complete unawareness of the stimuli (blindsight Type I) or awareness of stimuli of a non-visual sort (blindsight Type II). Some of these feats include indicating accurately the location of stimuli and even differentiating between static and moving stimuli.

In cognitive terms, blindsight is a particularly interesting case, given that subjects with this impairment “guess” correctly well above chance, indicating cognitive processing of the stimuli presented. But this is behaviourally less interesting than the fact that a person with blindsight might actually be able to make appropriate grasping movements towards objects presented in their blind field (Marcel, 1998) or navigate obstacles while moving in a room (de Gelder et al., 2008). In fact, this last phenomenon in particular compares favourably in behavioural terms, because there is no task imposed or forced upon them by an observer. On the contrary, the patient detours the obstacles without any assistance, thus showing cognitive autonomy regarding the practical task of skilfully walking in the middle of encumbering obstacles, an everyday situation that often causes accidents for people with no visual deficits. Concerning the study of de Gelder and colleagues (2008), it is important to note that the patient in question has a complete blind visual field due to bilateral damage to the striate cortex that spares no portion of it. The aim of the study was precisely to assess the visual capacities in the absence of V1, thus implicating an entirely extra-striate pathway of visual processing.

 It was more recently found that the cognitive states related to unconscious visual processing in blindsight might be of a higher level, involving the processing of meaning. In fact, Marcel (1998) found that patients were semantically biased to words presented in their blind fields. Also, by using conditioning techniques and covert responses, such as skin conductance responses (SCR), some studies (e.g., de Gelder, Vroomen, Pourtois, & Weiskrantz, 1999; Hamm et al., 2003) revealed that there is some processing, dubbed affective blindsight, of visual, emotionally charged stimuli (e.g., facial expressions) in blindsight patients. Again, an extra-striate pathway appears to account for this processing, namely the superior colliculus-pulvinar pathway to the amygdala, which is linked strongly to responses to emotional stimuli, especially to fear (de Gelder, Vroomen, & Pourtois, 2002; Liddell et al., 2005; see Figure 1). Although in this case no studies report more interactive, overt behaviour displayed by patients, the covert responses suggest that there is some form of cognitive processing with consequent formation of beliefs and intentions that are not fully realized in behavioural terms.^10^

 The main criticism against blindsight was launched by Campion and colleagues (Campion, Latto, & Smith, 1983). It comprised four main objections, one theoretical, and the other three of a more methodological character. In relation to the first, Campion and colleagues claimed that the use of forced-choice procedures is not compatible with the theory that the subjects are not conscious of the stimuli. Regarding the other three objections, the authors of the study suggested that blindsight is an effect of scattered light, spared cortex, and near threshold vision. Cowey (2004) attempts to address all these obstacles. More recently, Sahraie and colleagues (Sahraie, 2007; Sahraie et al., 2006) have rekindled debate by reporting increased visual sensitivity in field defects after repeated stimulation (training). This might suggest the existence of spared islands of conscious vision, namely spatial channels of processing. However, Ptito and Leh (2007) tested hemispherectomized patients with blindsight whose occipital lobe had been entirely removed or disconnected (deafferented) from the rest of the brain. This ruled out the existence of spared islands of visual cortex in the blind fields as an explanation for the presence of visual abilities (see also de Gelder et al., 2008). However, Cowey (2010) shows that the saga of blindsight is not yet over. 

###### Prosopagnosia

Covert responses is all we have so far regarding prosopagnosia, the inability to recognize individual faces, namely those of spouses, close relatives, and friends, and even one’s own. Clinically, the condition seems to be well defined. It appears to be associated with bilateral lesions involving the central visual system in the mesial occipitotemporal region (Damásio, 1985; Damásio, Tranel, & Damásio, 1990). The current trend is to see it as a memory impairment, namely the failure to activate memories relative to specific visual stimuli. In fact, prosopagnosia is often a specific inability within a more general failure to identify tokens of types of stimuli (e.g., clothes, fruits, vehicles, etc.) which the patients can recognize accurately (e.g., Damásio, 1985). The condition is not normally associated with a degradation of either other cognitive skills or complex visual abilities, except for a frequently observed acquired achromatopsia (Damásio et al., 1990).

Cognitively interesting is the finding in the early 1980s that the failure to recognize consciously the faces of familiar people is accompanied by a covert emotional arousal (SCRs; e.g., Bauer, 1984; Tranel & Damásio, 1985; for a double dissociation with bilateral ventromedial frontal damage, see Tranel, Damásio, & Damásio, 1995). This suggests that an unconscious recognition indeed takes place. The sparing of a dorsal visuolimbic pathway in the impairment of a ventral one is hypothesized to account for this unconscious recognition (cf. Bauer, 1984, p. 465).^11^ Although this may not appear to be so important in cognitive terms, these covert responses indicate successful matches between percepts and thus knowledge without awareness (Tranel & Damásio, 1985); experiments involving more directly observable behaviour would likely strengthen this interpretation.

Of direct interest for this survey is the rejection of a dorsal visuolimbic pathway to explain the unconscious arousal verified in patients with prosopagnosia when shown faces of familiar people (e.g., Breen et al., 2000). However, neurophysiological studies appear to have so far supported Bauer’s (1984) distinction of two visual pathways that may somehow involve the limbic system (thought – controversially; e.g., Kötter & Stephan, 1997, to be responsible for emotional arousal), or parts of it, or simply limbic structures. For instance, Tranel and the Damásios (1995) found a dissociation between bilateral occipitotemporal and bilateral ventromedial damage: Whereas the former impairs recognition but allows SCR discrimination, the latter impedes SCRs in the recognition of the identity of familiar faces. However, the fact that there seems to be a specific hereditary or congenital kind of prosopagnosia without apparent brain lesions or known malformations (Behrmann & Avidan, 2005; Grüter, Grüter, & Carbon, 2008) calls for more research into this condition, namely as far as covert responses to faces suggesting unconscious recognition are concerned.

###### Left visuo-spatial neglect

*Left visuo-spatial neglect*^12^ is the failure to perceive the left visual side of space. Patients consistently neglect whatever item is on their left, leaving food on the left side of their plates, shaving or making-up only the right side of the face, dressing only the right part of their bodies and wholly neglecting the left side of both their bodies and their clothes, writing only on the right side of a sheet of paper, etc. (cf. Halligan & Marshall, 1998, for abundant examples). Anatomically, left visuo-spatial neglect is associated with damage to the right hemisphere, specifically to inferior parts of the parietal lobe at the temporoparietal junction (e.g., Vallar, 2001), probably implicating the ventral stream (McIntosh et al., 2004; Schindler et al., 2004; but see Singh-Curry & Husain, 2009). That we can speak of *left visuo-spatial neglect* almost as a synonym for *unilateral neglect* is due to the rarity of neglect of the right visual side, attributed to compensatory or redundant processing of the right side of space by both cerebral hemispheres.

 The dubbing of the condition as neglect, however, is a cause of annoyance to the patients, who claim that there is simply no left side of space (Halligan & Marshall, 1998, p. 358) and can thus be seen as anosognosics. In fact, and especially in cases involving other modalities, this failure to acknowledge the left side may go as far as a denial of ownership of one’s left part of the body (e.g., Cutting, 1978). Unsurprisingly, when asked to identify stimuli on the left side of space, they have very poor results, or fail completely. However, this is at odds with the fact that they perceive and process those stimuli, even at a semantic level. For instance, Berti and Rizzolatti (1992) reported facilitation of responses with semantic priming (highly congruent or congruent stimuli presented to the neglected field simultaneously with clearly perceived visual targets in the unaffected visual hemifield). In addition, showing semantic processing on a higher behavioural level (decision making), Marshall and Halligan (1988) reported that a patient with severe visual neglect consistently chose the line drawing of an intact house as compared to a line drawing she was simultaneously presented with of the same house with its left side on fire, though she claimed that the houses were identical. 

If we side with James (1904; see introductory quotation) in the belief that knowing is a way of establishing fruitful relations with reality, that is, knowledge is just successful behaviour, then the next finding is an example par excellence of unconscious knowledge. As already noted, obstacle avoidance when reaching for objects or moving in space requires good knowledge of the workspace. It has been reported (McIntosh et al., 2004) that patients with left visuo-spatial neglect, when asked to reach between obstacles they cannot discriminate, take such obstacles into account in their trajectories. In contrast, when asked simply to point midway between two stimuli, their performance is frankly poor. This automatic avoidance of obstacles is attributed to the dorsal stream, in this and other forms of visual agnosia (e.g., Rice et al., 2006; Schindler et al., 2004; Striemer et al., 2009).

Recent studies have challenged the localization of the lesions contributing to this condition, namely as far as the inferior parietal lobe is concerned (e.g., Husain & Rorden, 2003). As a matter of fact, it appears that it can be caused by lesions in many areas of the brain (Vallar, 2001; Vallar & Perani, 1986), which can explain the heterogeneity of the condition.

##### Perception under general anaesthesia and in coma

In common, *general anaesthesia* and coma have the apparent complete unresponsiveness to stimuli, namely the inability to wake up under stimulation. In both cases, the levels of arousal or wakefulness and of awareness are minimal or zero, and though we do not yet fully understand them in anatomical, neurochemical, and physiological terms, the similarities between the two states allow us to speak of general anaesthesia as an induced and controlled coma (e.g., Bleck, 2002). Research into unconscious perception under anaesthesia and in coma states presents very practical objectives. The question as to whether patients undergoing surgery under general anaesthesia somehow cognitively process the surrounding environment (medical personnel speaking in the operating room, for instance) might be relevant in terms of the avoidance of traumatic experiences or, on the contrary, for the improvement of postoperative healing. As for cognitive processing in states of coma, it might help predict patient survival. However, the overall evidence in favour of cognition in states of anaesthesia was, until recently, scarce or outright conflicting, which calls for more research (for reviews, see Andrade, 1995; Kihlstrom & Cork, 2007). The case of coma, for obvious reasons, is more difficult to study experimentally in terms of cognitive processing; perhaps the more secure results we have so far are based on event-related potentials (ERP; e.g., Daltrozzo et al., 2009; further literature below).

###### General anaesthesia

Research in this particular field was sparked largely by a study (Levinson, 1965) which showed that patients who had undergone surgery with general anaesthesia could remember, under hypnosis, a conversation among the medical personnel indicative of an anaesthetic crisis concerning them. Besides hypnosis, Levinson also provided support with electroencephalograms (EEG) recorded during the surgery that showed an augmentation of high-voltage slow waves coinciding with the anaesthetist’s words “Just a moment! I don’t like the patient’s colour. Much too blue. His (or her) lips are very blue. I’m going to give a little more oxygen.” This change in the EEG only subsided minutes after the tranquilizing final words “There, that’s better now. You can carry on with the operation” (Levinson, 1965, p. 544), and it was present in the EEGs of even those patients who did not recall the event under hypnosis.

Levinson reported the interesting finding that, 1 month after the operation, under hypnosis, of the ten patients involved in the experiment, four repeated almost literally the anaesthetist’s words and another four vaguely remembered hearing someone talking – some of them were even capable of identifying the anaesthetist. Levinson’s experiment presents several methodological problems, not the least of which is the use of hypnosis.

In fact, hypnosis, a highly variable and still-misunderstood phenomenon (cf. Kihlstrom, 1985), was largely abandoned as a means to test memory of an event occurring in a state also not fully understood (e.g., Evers & Crowder, 2009). More recently, the application of indirect or implicit measures (de Houwer, 2006; Merikle & Reingold, 1992; Reingold & Merikle, 1988) including word-association (Kihlstrom, Schacter, Cork, Hurt, & Behr, 1990), familiarity judgments (Jelicic, Bonke, De Roorde, & Bovill, 1992), and preference judgments (Block, Ghoneim, Sum Ping, & Ali, 1991) helped to report unconscious processing of stimuli presented during anaesthesia. In the first study (Kihlstrom et al., 1990), patients under anaesthesia were played several times (an average of 67 repetitions), through earphones, a list of 15 paired associates (e.g., *ocean-water*). In the recovery room, when the patients were asked to respond to a cue with the first word that came to mind, they were more likely to produce the targeted response from the list, compared to targets of a control list. This result suggests that they had unconsciously perceived the stimuli, a conclusion further strengthened by the fact that the patients performed badly in a task of explicit recall (see below the Implicit Memory section). Jelicic and colleagues (1992) reported that patients who had been exposed to a list of fictitiously famous people were more likely than another group to designate more non-famous names as being famous, also suggesting unconscious auditory perception and processing of the information acquired. The study of Block and colleagues (Block et al., 1991) reported results that indicate not only that unconscious cognition takes place during anaesthesia, but also that this may be independent of the method of anaesthesia employed.

Despite these results, the phenomenon of perception during anaesthesia is strongly contested, not only because of the anti-intuitive nature of the theory, but also – and mainly – because of the lack of (more) robust results. As a matter of fact, other studies applying the same measures as those above failed to replicate their results (for reviews, see Andrade, 1995; Caseley-Rondi, Merikle, & Bowers, 1994; Merikle & Daneman, 1996).

###### Coma

We have reason to believe that *coma* is characterized by total unresponsiveness to stimuli, both internal and external, as comatose patients show no evidence of awareness either of self or of the environment, remaining in an unvarying eyes-closed state even under intense stimulation. This, together with other neurophysiological measures, allows us to see coma as a state of absence of both arousal and awareness, and thus as a radical dysfunction of consciousness (Posner, Saper, Schiff, & Plum, 2007). It is commonly caused by severe brain injury involving relatively discrete bilateral subcortical structures or diffuse injuries in both hemispheres to both subcortical and cortical structures (Schiff, 2007). It can evolve into either fast recovery or a plethora of highly dysfunctional states, such as vegetative state, locked-in syndrome, and even brain death (Laureys, Owen, & Schiff, 2004).

 Given this clinical picture involving so many issues (medical, ethical, etc.), it is only legitimate that we should want to know whether there is any cognitive processing taking place in this condition, namely for prognostic ends. However, more than any other condition, this poses particularly difficult problems concerning measures of cognitive processing, as it is characterized as a state of no consciousness (Laureys et al., 2004). On the other hand, this makes it a privileged candidate for studies in unconscious knowledge, for, as consciousness is ruled out, any mentation taking place can be more securely considered unconscious. Event-related brain potentials are believed to relate to psychological demands (attention, memory, etc.) *invoked* by a situation rather than merely reflecting brain activity strictly *evoked* by the presentation of a stimulus, that is, basic sensory processes (*evoked* potentials; e.g., Rugg & Coles, 1995). These ERPs provide an invaluable method to have a glimpse of higher mental processes in coma. For instance, Reuter and Linke (1989) recorded the P300, a late auditory ERP component, in coma patients who survived. This is a finding confirmed by subsequent studies (e.g., Gott, Rabinowicz, & de Giorgio, 1991). The components P300, and particularly N100 and mismatch negativity (MMN), a response to a deviant stimulus in a series of regular stimuli, have been confirmed as reliable predictors of recovery from coma by a number of studies. This confirmation indicates that the evaluation of ERPs should be performed in the prognosis for the awakening of comatose patients (e.g., Luauté, Fischer, Adeleine, Morlet, & Boisson, 2005). 

Daltrozzo and colleagues have recently conducted an experimental study to evaluate cortical information processing in coma using ERPs (Daltrozzo et al., 2009). The study is particularly interesting for this survey, as it also concerns semantic processing and does so within the priming paradigm, thus having an immediate connection with the next section (see Masked Priming section). Briefly, *semantic* priming is the activation of the processing of the meaning of words by means of the presentation of stimuli, typically words (primes). In specific forms of unconscious semantic priming, the primes cannot be identified (e.g., they are masked; see next section for details). In Daltrozzo and colleagues’ (2009) study, the subjects were patients in acute non-traumatic coma with a Glasgow Coma State (GCS) < 9 (Teasdale & Jennett, 1974). Daltrozzo and colleagues presented them with 120 word pairs (the word pair priming paradigm), 60 were semantically related and 60 were unrelated, and 100 sentences (the sentence priming paradigm), 50 with congruent and 50 with incongruent end words. Responders were found for both semantic paradigms (seven for the word pair paradigm and three for the sentence paradigm) and their distribution was statistically different from that expected by chance. More specifically, the N400, a component of ERPs connected to the processing of meaningful stimuli, was elicited in both paradigms by target words when semantic incongruity was involved, replicating findings in normal subjects (Kutas & Hillyard, 1980), namely within the priming paradigm used in studies of unconscious perception (e.g., Kiefer, 2002). In light of these results, the authors questioned the assumption that high-level mental processes require explicit, conscious processing.

##### Masked priming

One possible definition of *priming* is the activation, by means of sensory input, of stored information, making it more available to a person and thus influencing their perception and thought processes. When this influence is negative, actually inhibiting these processes in some way, as for instance in the Stroop interference effect, this is called *negative priming* (see Tipper, 2001, for a review). In a typical experiment, two stimuli are presented successively, the target following the prime. Take a disambiguation task, for instance, in which the word *river* (the prime) is presented before the word *bank* (the target).^13^ We say there has been priming when the prime facilitates response to the target, in this case, when in reading the semantically ambiguous sentence, *They stood by the bank*, the subject interprets bank as *riverside* instead of as *financial institution*. Given that the prime is supposed to be unattended to, this phenomenon is particularly interesting for studies in unconscious cognition, being directly connected to experiments and theorizing on subliminal perception. This therefore provides a continuity between earlier experimental psychology (see Unconscious Cognition from Early Experimental Psychology to Cognitive Psychology section) and contemporary (neuro)cognitive research. As a matter of fact, we can say that it has contributed enormously to the current wider acceptance of unconscious perception, having propelled much fruitful debate concerning most aspects of unconscious mentation (Kouider & Dehaene, 2007) and being especially connected to the topic of implicit memory (see Implicit Memory section).

This is so because priming is theorized to occur in the absence of conscious perception of stimuli. More specifically, in the case of stimuli below certain thresholds – intensity, duration, etc. – it is thought to take place only on an unconscious level. While there are many priming methods, masked priming is particularly interesting. This is where typically a mask (commonly a string of symbols: e.g., “#####”; scrambled patterns, or letters; see Kunde, Kiesel, & Hoffmann, 2005, and Van Opstal, Reynvoet, & Verguts, 2005, for a debate on the importance of the type of mask) is presented immediately before the prime (forward masking), after it (backward masking), or simultaneously with it (simultaneous masking). This is an interesting method of testing unconscious perception because it is believed that it precludes conscious perception of the relationship between the prime and the target by masking the prime, that is, by wholly hindering its detection and recognition (e.g., Forster, Mohan, & Hector, 2003). Moreover, it can be applied to various stimulus-response situations, from visual (for reviews, see Ansorge, Francis, Herzog, & Öğmen, 2007; Breitmeyer, 2007) to auditory stimuli (Dupoux, de Gardelle, & Kouider, 2008; Kouider & Dupoux, 2005), eliciting processes ranging from motor responses to semantic representations. It is thus not surprising that research into masked priming is an extensive and extremely active field, now developing into a large number of approaches and theories. These include the sensorimotor supremacy hypothesis (Ansorge, Neumann, Becker, Kälberer, & Cruse, 2007), masked face priming (Henson, Mouchlianitis, Matthews, & Kouider, 2008), event-related potentials in priming paradigms (Kiefer, 2002; Kiefer & Brendel, 2006), the concept of direct parameter specification (Ansorge & Neumann, 2005; Neumann, 1990), etc. While response priming, involving motor responses, has long been a well accepted phenomenon, the results of semantic priming were recently questioned (e.g., Abrams & Greenwald, 2000; Damian, 2001), making it a central point of debate within studies of unconscious knowledge for much of this decade. For this reason (as well as for lack of space) and especially because it is connected both to implicit memory (a topic discussed in this paper) and to unconscious processing of meaning, suggesting that unconscious processing of information can be extended to higher levels of mental activity, this section will focus on masked semantic priming.

The necessarily brief discussion will be restricted to a study that, though not recent, is still illustrative of the impact of masked semantic priming on the topic of unconscious knowledge, having moreover motivated much of the ongoing research. Inspired by clinical phenomena, such as blindsight (see above) and deep dyslexia, in which there appears to be a dissociation between perceptual processing and the ability to verbalize and/or voluntarily use the results of that processing, A. J. Marcel carried out a set of five experiments with visual masked priming (Marcel, 1983a; see Marcel, 1983b, for a theoretical discussion). These experiments aimed to counter what he called the *identity assumption*, according to which the representations of conscious experience are the same ones that are derived and used in sensory and motor processes. Of the five experiments, three (Experiments 3, 4, and 5) are of particular interest for us, though they are all interconnected in some way. In Experiment 3, unconscious semantic (or at least lexical) processing was believed to have been verified when subjects identified manually colour patches that were either accompanied by or preceded by masked words. When the words were colour-incongruent, they delayed reaction time (RT), in a Stroop-like effect, whereas colour-congruent words facilitated RT when pressing the button corresponding to the presented colour patch. Marcel drew some important conclusions from these results, the most important for us being that a masked word, which is not only unreportable but also undetectable, can be semantically represented.^14^ Experiment 4 was actually conceived to compare results between central pattern and peripheral energy masking. However, the result that interests us is whether RT (in deciding whether or not a string of letters is a word; a lexical-decision task) was speeded up if the (central pattern-)masked string was a word related in meaning (e.g., *child-infant*), a result that corroborates the conclusion of Experiment 3. With Experiment 5, Marcel verified that repeating a word-plus-mask from 1 to 20 times increased the association effect, whereas it had no effect on detectability or the semantic relatedness of forced guesses of the masked word, that is, “detectability and awareness could not be built up” (Marcel, 1983a, p. 229). This result goes against the argument that the priming effect depends merely on the amount of stimulus information required for awareness. According to Marcel, this suggested that while pattern masking leaves intact a representation mediating an accumulation of lexical and/or semantic priming, it does not leave intact anything that mediates accumulation of whatever it is that is necessary for a conscious representation; this rules out semantic activation, no matter how strong, as mediating consciousness. The results, especially of Experiments 3, 4, and 5, suggested to their conceiver that non-conscious representations should be investigated by looking at the way they influence behaviour rather than by asking subjects to undertake the “phenomenally bizarre” (Marcel, 1983a, p. 212) task of selectively using inaccessible representations. In other words, unconscious perceptual processing should be measured indirectly (Destrebecqz & Peigneux, 2005; see Coma section for further literature).

 In spite of the fact that a number of studies replicating all or some of the results of Marcel’s experiments immediately followed (Marcel, 1983a, p. 232), criticism concerning methodological and theoretical issues was soon published, questioning the unconscious status of the unreportable words (Cheesman & Merikle, 1984; Holender, 1986). This criticism prompted much research and the integration of stronger experimental techniques, from brain-imaging (Dehaene et al., 1998) to methods allowing an easier replication of the results (Draine & Greenwald, 1998). As was mentioned above, semantic priming had to face further and more recent challenges, particularly after studies conducted by Abrams and Greenwald (2000). They argued that masked primes are analysed mainly at the level of word parts and not as complete words, thus questioning the processing of meaning. Damian (2001) raised the fundamental question of whether semantic priming, instead of the unconscious semantic processing of subliminal information, merely reflects automatized stimulus-response mappings. These alternative explanations appear to have been reliably ruled out by recent studies (e.g., Kiefer, 2002; Kiefer & Brendel, 2006; Kiefer, Martens, Weisbrod, Hermle, & Spitzer, 2009), but the extent to which the processing involved is wholly unconscious, a concern that goes right to the heart of this topic, remains debatable (e.g., Kouider & Dehaene, 2007; Kouider & Dupoux, 2004).

#### Unconscious Learning and Retrieval

The postulations of an unconscious or implicit memory system and of unconscious or *implicit learning* are not easily distinguishable: In general terms, implicit learning is the ability to acquire knowledge that is not reportable, or is only reportable with difficulty and imperfectly. *Implicit memory* is the memory that affects behaviour and judgements without the subject being able to intentionally recall it. In other words, implicit learning is the non-intentional and incidental acquisition of information about structural relations between objects or events, whereas implicit memory is the non-intentional recourse to a prior learning episode in the performance of a more or less related task (cf. Buchner & Wippich, 1998; see also Dienes & Seth, 2010). In this context, unconscious knowledge is the information that is acquired in an unconscious way and/or stored in a memory system largely or completely inaccessible to consciousness. For instance, most people are incapable of describing most of the grammatical rules they use when speaking their native language, a particularly striking phenomenon in the case of very young (less than 5-6 years of age) fluent speakers not yet acquainted with any notions of grammar. A domain in which we often make use of unconscious knowledge is that of social psychology. We can quickly (mis)judge people by drawing on often-quickly-formed attitudes and stereotypes without being aware of that fact and even less so of the rules and constructs we apply in those instances (e.g., Downs & Lyons, 1991; Lewicki, 1986; for a review, see Steele & Morawski, 2002). Important paradigms in the field of implicit learning have been the use of artificial grammars, which is also applicable to research in implicit memory, namely in cases of impaired memory, and of simulated complex systems. Another approach of interest is learning during sleep.

##### Artificial Grammars and Simulated Systems

**Figure 2.**
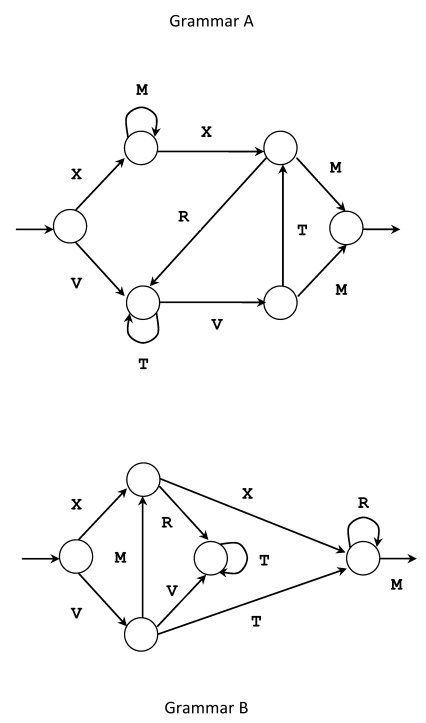
Examples of Artificial Grammars. Grammar A originates the following strings: xmxrttvtm, vttvtrm, xmmxrvm, vtvtm, xxrvtm, etc. Adapted from “Unconscious Knowledge of Artificial Grammars is Applied Strategically” by Z. Dienes, G. T. M. Altmann, L. Kwann, and A. Goode, 1995, *Journal of Experimental Psychology: Learning, Memory, and Cognition, 21*, p. 1323; and from ”Transfer of Syntactic Structure in Synthetic Languages” by A. S. Reber, 1969, *Journal of Experimental Psychology, 81*, p. 116 (Grammar A).

Research into implicit learning with artificial grammars was initiated by A. S. Reber in the late 1960s (Reber, 1967, 1969) and sparked an abundant literature on this phenomenon. This abundance reflects the complexity of the overall claim that, exposed to strings of sentences produced by artificial grammars (see Figure 2) without a learning strategy, subjects actually acquire unconscious knowledge of the grammars. To support this claim, there is the finding that subjects in this experimental paradigm can distinguish grammatical from non-grammatical strings well above chance, while showing no confidence regarding this skill and being incapable of verbalizing their knowledge of the grammars:

When subjects said they were literally guessing, they were in fact performing significantly above chance with a classification performance of 65% (*SD* = 20%), *t*(9) = 2.31, *p* < .05. That is, subjects did not know that they knew. Further the slope of the regression line was non-significantly different from zero, *F* < 1. That is, subjects did not know when they were in different knowledge states. On both these grounds, the knowledge is attitude implicit. (Dienes & Perner, 2003, p. 229)

Relevant to this paradigm is the fact that the grammars are too complex to be learned consciously even over a long period of time. In addition, they are not necessarily alphabet-based: For instance, shapes, colours, etc., can be used, namely in experimentation on transfer of unconscious knowledge across modalities (e.g., Altmann, Dienes, & Goode, 1995; Manza & Reber, 1997).

In addition, making use of complex rule systems and appealing to the capacity of subjects to acquire unconsciously knowledge of those systems, is the research initiated in the late 1970s by Broadbent and colleagues (Berry & Broadbent, 1984; Broadbent, 1977; Broadbent & Aston, 1978; Broadbent, FitzGerald, & Broadbent, 1986). Their studies aim to show that correct performance on a control task (reaching and maintaining specified target values by varying a single input variable)^15^ does not depend on the capacity of subjects to verbalize either knowledge of the systems they are asked to control or how they manage to control them successfully. Although the subjects’ performances improve with practice, this is not mirrored in an improvement in the capacity to answer questions about the workings of the system. Contrastingly, verbal instructions given to subjects improve their ability to answer questions, but have no import for their actual performance in controlling the systems. Given that this control, in requiring sustained performance, is carried out very much like a manual skill, this strongly corroborates the hypothesis that we physically act securing success in situations in which the sole knowledge we can make use of is of the unconscious kind.

Perhaps more than any other field in which unconscious knowledge is involved, experimentation with artificial grammars has faced important challenges; these date from its inception and continue today with certain regularity. This is not surprising, given that, as Dulany and colleagues (Dulany, Carlson, & Dewey, 1984) say:

Nowhere is the claim for unconscious processes stronger, or more significant if true, than when the hypothetical processes are among the most complex of which we are capable – processes such as abstraction, inference, decision, and judgment. This is the claim for a fully psychological unconscious. (p. 541)

The first major challenges were launched by Dulany and colleagues (Dulany et al., 1984) and targeted the initial results obtained by Reber (1967, 1969, 1976), as well as further developments (e.g., Allen & Reber, 1980). Though their experimental results roughly replicated Reber’s, they concluded that implicitly instructed subjects showed no more learning than those explicitly instructed; moreover, according to them, the learning verified in the former could be accounted for by the subjects’ consciously learning the rules, namely by acquiring correlated grammars. A controversy ensued over methodology rather than over the distinction between conscious and unconscious knowledge (Brody, 1989; Dulany, Carlson, & Dewey, 1985; Reber, Allen, & Regan, 1985).

However, the challenges faced by the theory of unconscious learning in relation to artificial grammars greatly stimulated its development and polished the methods used for testing and measuring (for a review, see Destrebecqz & Peigneux, 2005). For instance, forced-choice tasks appear to be well suited to elicit implicit knowledge (as compared to free reports and questionnaires). Important developments were the distinction and definition of *objective* and *subjective thresholds* (Cheesman & Merikle, 1984) and the devising of subjective measures, such as the guessing and the zero-correlation criteria (Dienes, 2007; Dienes et al., 1995). Briefly, perception is said to be under the *subjective threshold* when the subjects identify a target above chance performance while claiming not to have perceived it. We say that subjects are under the *objective threshold* when identification is at chance level, from which it is concluded that the target was simply not perceived. It is when under the subjective threshold that we say that a subject lacks metaknowledge, that is, they do not know that they know. Subjective measures ask subjects to report their mental states, and not just to discriminate stimuli: They measure the extent to which subjects think they know (vs. how much they actually know). So far, two important criteria of subjective measures have been established: These correspond with two ways in which this lack of metaknowledge can be expressed and measured, the guessing criterion, measuring to what extent a subject’s belief that they are only guessing is contradicted by performance on a task, and the zero-correlation criterion, measuring the lack of a correlation between a subject’s confidence and accuracy in the tasks. In the first case, unconscious knowledge is claimed to account for the contradiction between the subject’s belief that they are merely guessing and the above-chance performance, and, in the second case, unconscious knowledge is said to be demonstrated when subjects are equally confident in both accurate and inaccurate decisions.

Research in the artificial grammars paradigm has more recently received contributions from ERP-based studies looking for neural correlates of the cognitive demands involved (e.g., rule adherence, chunk formation, etc.) in unconscious grammar learning (e.g., Lieberman, Chang, Chiao, Bookheimer, & Knowlton, 2004; Skosnik et al., 2002). An interesting finding concerning neural correlates in the artificial grammar paradigm is a dissociation found by Seger and colleagues (Seger, Prabhakaran, Poldrack, & Gabrieli, 2000) between the neural bases of implicit and explicit learning.

##### Implicit memory

**Figure 3.**
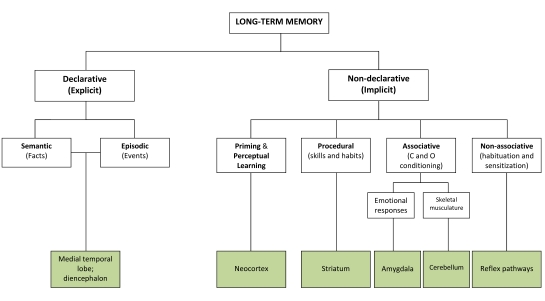
The Multiple Memory Systems Approach. “Associative” and “non-associative” refer to forms of learning. Adapted from “Cognitive Neuroscience and the Study of Memory” by B. Milner, L. R. Squire, and E. R. Kandel, 1998, *Neuron*, 20, p. 451.

As seen, experimental psychology was, from its beginnings, very much interested in unconscious processes, but until recently, it failed to unite the fields of unconscious memory and unconscious learning. As a matter of fact, while the terminological distinction between implicit and explicit kinds of memory and knowledge dates from as early as the 1920s (McDougall, 1924), talk of implicit learning began properly only with A. S. Reber in the late 1960s (Reber, 1967), though studies on unconscious learning processes had started long before this coinage (e.g., Thorndike & Rock, 1934). A few years after Reber’s first experiments with artificial grammars, psychology saw the explosion of a plethora of distinctions of memory systems (see Sherry & Schacter, 1987, p. 446), of which only a few became more or less orthodox; in particular, the contemporary major distinction of the multiple memory systems approach (see Figure 3) is widely accepted.

This distinction, contrasting declarative vs. non-declarative memory systems, is of clear interest for this survey in that it largely equates with the already known explicit versus implicit dichotomy, now applied to memory systems. It also incorporates elements of the widely-accepted distinction between semantic and episodic memory subsystems (Tulving, 1972) that might help to clarify the concept of *declarative* in knowledge issues. *Declarative memory*, as the name implies, is memory that can be verbalized, hence brought to consciousness and explicit in the sense that one is aware of it. It is believed to comprise two subsystems, *episodic memory*, a storage system of events, and *semantic memory*, a storage system for words and concepts. As for *non-declarative memory*, its main characteristic is that, contrary to declarative memory, it is not easily (if at all) verbalizable, remaining implicit and observable only in behaviour. According to this approach, it comprises subsystems for priming, associative learning, and non-associative learning, together with procedural memory, in this view, specifically reserved for skills and habits. Supporting this functional distinction are findings in neuropsychology indicating different cerebral localizations (e.g., Atallah, Frank, & O’Reilly, 2004; Bechara et al., 1995; McDonald & White, 1993).

An important study (Berry & Dienes, 1991; see Buchner & Wippich, 1998, for a criticism and caveats) puts implicit memory and implicit learning into relation with respect to key features and underlying pro-cesses. It concludes that, though independent to some extent, there is the possibility of a common field of research in that the same processes seem to underlie performance in tasks involving the two phenomena. In fact, they verified that, by and large, both implicit memory and unconscious learning/knowledge are (a) tied to the surface characteristics of stimuli, (b) more durable than their conscious counterparts, (c) less affected by variables manipulation of the level or type of study processing, (d) partly stochastically independent (see Tulving, 1985) of their conscious counterparts, and (e) unaffected by amnesia.

Key feature (e) is extremely important for the dichotomy at issue in that it strongly corroborates the view that unconscious learning and implicit memory are indeed different phenomena from conscious learning and explicit memory, and not merely knowledge or memory that fails to reach consciousness, but could do so. Briefly, neurological amnesia, following lesions to the medial temporal and diencephalic regions of the brain in cases like head injury, anoxia, chronic alcohol abuse (Korsakoff amnesia), ischemia, etc., is the inability to remember past events and facts (*retrograde amnesia*) and/or new information (*anterograde amnesia*) in the normal functioning of the other perceptual and cognitive faculties. This appears to indicate that amnesia is an impairment of explicit or declarative memory alone (see Figure 3). In fact, studies in amnesia have shown that, while amnesic patients are seriously impaired in tasks of explicit memory (recall, free or cued, and recognition; e.g., Squire & Shimamura, 1986) and in recollecting past facts and events (e.g., Reed & Squire, 1998), they performed well in tasks involving implicit memory, such as repetition priming and skill learning (Graf et al., 1984).

Concerning the first tasks, repetition priming is the facilitation in the processing of a stimulus owing to a recent encounter with that stimulus (see above). This facilitation is verified, for example, in decreased latencies in the making of lexical decisions and in a tendency to use words to which one has been exposed in tasks such as word completion. The study by Graf and colleagues (Graf et al., 1984), based on an activation account of memory,^16^ is of particular importance in that it contrasts results obtained by three kinds of amnesic patients in tasks of explicit and implicit memory. Whereas all patients in the study were clearly impaired by comparison to control subjects in tasks of explicit memory, they performed normally in a task of word completion. Tasks involving skill (motor and cognitive) learning in amnesic patients also appear to indicate that there is implicit learning in the absence of any conscious memory: For instance, amnesic patients have shown progresses in mirror-tracing tasks (Milner, 1962), and rule learning has been verified (Kinsbourne & Wood, 1975).

None of the many studies in implicit memory in amnesia, however, shows in such a vivid way and in cognitive-behavioural terms that we can speak of an unconscious memory system as the “experiment” carried out by Claparède (1911/1995). He deliberately pricked a profoundly amnesic patient with a pin hidden in his hand when shaking hands with her; following this event, the patient, albeit unable to consciously remember it, refused to shake hands with him, claiming that it was well known that sometimes people hide pins in their hands.

For a long time, research in impaired memory has directly applied the artificial grammars paradigm with results that support the above. For instance, we have known for some time that amnesic patients perform normally in indirect measures of implicit grammar learning (e.g., Knowlton, Ramus, & Squire, 1992). More recently, using this paradigm with patients with early Alzheimer’s disease, who often exhibit impaired declarative memory, Reber and colleagues (Reber, Martinez, & Weintraub, 2003) found evidence suggesting that implicit memory formation was intact.

 This field of research is not without its challenges; inevitably, the main issue involves the possibility of confusing implicit with explicit memory, namely with involuntary explicit memory (see Schacter, 1987, p. 510). Contrary to research with artificial grammars, this field has not carried out a significant assessment of the methodology used in the experiments, perhaps due to the fact that it has not sustained such vigorous and repeated challenges as the former, though such challenges exist and call for such an assessment. For instance, Buchner and Wippich (2000) argue that a reliability difference (implicit memory tests commonly have low reliability vs. high reliability of explicit memory tests) might be behind the results suggesting a dissociation between the two kinds or systems of memory. One of the reasons for the lack of such active opposition as that encountered by research into implicit learning is that the theoretical distinction between implicit and explicit memory is not only apparently verified by experimental behavioural studies (again, see Buchner & Wippich, 2000), but also by physiological approaches which strongly suggest that these memory systems are anatomically distinct (e.g., Buckner at al., 1995; Voss & Paller, 2007; cf. Figure 3). Moreover, the findings discussed above (that patients with impaired memory exhibit normal levels of performance in tasks of implicit memory) also support the distinction. 

##### Learning during sleep

A long-standing interest in learning during sleep is due to the high degree of unresponsiveness in the otherwise apparently normally functioning perceptive and cognitive apparatus:^17^ just how much cognition can actually take place in this state characterized by a reduction of exteroceptive stimulation? Learning during sleep is so appealing to so many that it has actually become a whole industry, namely in language learning.

However, the evidence in favour of cognition in states of sleep was for a long time scarce, barely impeding an outright dismissal (Aarons, 1976). The stimuli presented in order to test the hypothesis of learning during sleep were of an auditory nature, for obvious reasons; recognition and recall (stimulated, unaided, guessing) were the most common testing methods (see Aarons, 1976, pp. 4-5). As said earlier, there was not much positive evidence^18^ and what scarce evidence did exist pointed to some learning coinciding with the appearance of alpha wave activations that occur more frequently during low-voltage EEG sleep, that is, rapid eye movement (REM) sleep, and thus closer to a state of wakefulness than to deep sleep (Aarons, 1976; Simon & Emmons, 1956; Tani & Yoshii, 1970). The fact that many studies simply did not include EEGs also helped to discredit research in this field.

More robust evidence suggesting that sensory stimuli are given some processing during sleep was possible with a more consistent neurocognitive approach facilitated by theoretical and technical advances in brain imaging (for examples of earlier studies, see Antrobus, 1990; Kutas, 1990; for a recent review, see Ibáñez, Martín, Hurtado, & López, 2009). More recently, studies in this line have included infants, due to the curious fact that, despite the long hours they spend sleeping, large amounts of learning appear to take place (e.g., Tarullo et al., 2010; Fifer et al., 2010). This research is in line with studies on memory consolidation during sleep (e.g., Brawn, Fenn, Nusbaum, & Margoliash, 2008; Stickgold, 2005; Stickgold, Hobson, Fosse, & Fosse, 2001). This, however informative, should not replace the need for studies more directly – though using indirect measures! – testing knowledge acquired during sleep, even because our understanding regarding the neural correlates of information processing, conscious or unconscious, is far from robust.

#### Unconscious Thinking and Decision Making

Decision making is of interest to studies in unconscious knowledge for the behavioural aspects it presents: Is one always aware of one’s decisions? What unconscious factors determine decision making? How do decisions taken unconsciously compare with conscious ones?; etc. Perhaps better than any other behavioural responses, decision making reflects the way information is gathered selectively and processed with a view to the wellbeing of the individual, as it involves such complex issues as “rationality” and “logicality,” aspects that are more often than not explicitly equated with consciousness. However, ongoing research into this topic suggests strongly that “rational” and “logical” thinking, or processing of information contained in a human knowledge base, can be carried out in a largely or wholly unconscious way: Both the somatic marker hypothesis and unconscious thought theory, without directly aiming to contribute to a theory of unconscious knowledge, offer it important material.

##### The somatic marker hypothesis

The somatic marker hypothesis (SMH; cf. e.g., Bechara & Damásio, 2005; Damásio, 1996) is the scientific way of putting what in everyday language we call “gut feeling,” a sort of “embodied knowledge” we cannot explain or specify but on which we are quite willing to ground our actions. As its name indicates, it postulates a crucial role to somatic states (emotional changes) in cognition, namely in decision making processes. In essence, it is the claim that cognitive states are associated with somatic changes that arise in bioregulatory processes, and that these associations, once stored in memory, are recalled in contexts similar to the one in which they first occurred. More specifically, the SMH sees the ventromedial prefrontal cortex (VMPFC) as the area of the brain where an association – a dispositional marking – between factual knowledge and bioregulatory processes is cognitively processed, that is, learned and stored (Bechara, Damásio, & Damásio, 2000; Damásio, Tranel, & Damásio, 1991). This marking is dispositional in that, once established, a situation similar to the original situation in which the association was first formed triggers a disposition for the same type of emotion, which, however, does not necessarily reactivate the same somatic states (the body loop), more often than not actually bypassing the body (the as-if body loop). Damásio and colleagues hypothesize that patients with damage to the VMPFC are impaired in learning this association, namely in cases in which somatic states mark situations involving punishment and reward. In fact, the hypothesis arose from the observation that people with lesions in the VMPFC showed disruptions in social behaviour in the absence of any intellectual and cognitive impairment. This disruption was especially noticeable in often-disastrous post-lesion decision making shown by the patients (cf. Damásio, 1994, for a full account of the famous case of Phineas Gage).

The main paradigm in the experimental study of this hypothe-sis is the Iowa Gambling Task (IGT; Bechara, Damásio, Damásio, & Lee, 1999; see also Bechara, Damásio, Damásio, & Anderson, 1994). This is a card-selection task involving four decks, two “good” decks, C and D, resulting in an overall gain in the end (despite low-paying individual cards), and two “bad” decks, A and B, resulting in a greater loss than the “good” decks (despite higher-paying cards). Damásio and colleagues have shown that patients with VMPFC damage perform poorly compared to normal subjects. They explain this result with the hypothesis that while normal subjects make decisions to some extent relying on anticipatory somatic markers (SCRs), the former cannot rely on such help. It is not the case that these subjects cannot produce SCRs when punished or rewarded: rather, they simply fail to produce the anticipatory SCRs that experience triggers in normals.

While these results provide corroborating evidence regarding patients with damage to the VMPFC, it is mainly the data obtained with normal subjects that are of interest to the theory of unconscious knowledge. In fact, it was found that before entering a period in which these subjects started to develop a hunch concerning what was going on in the IGT, they already produced higher anticipatory SCRs before selecting cards from the disadvantageous decks. Moreover, the 30% of normal participants who failed to reach a conceptual period (awareness of what was going on in the game) performed advantageously all the same. Damásio and colleagues actually implicitly invoked unconscious knowledge in these two cases, seeing the SCRs as unconscious biases guiding the decisions made by the subjects. Also of interest is the fact that they make a qualitative distinction based on the overt or covert processing of the somatic markers: if overt, they influence cognition at a conscious level; when covert, they contribute by biasing the cognitive process (e.g., Damásio, 1996, p. 1415). The contribution of the SMH to the field of unconscious cognition has been emphasized more recently (e.g., Bechara & Damásio, 2005).

 If there is a controversial theory today, it is the SMH (for a review, see Dunn, Dlagleish, & Lawrence, 2006); however, many of the challenges it faces are of no interest to this study, as they do not directly regard the issue of unconscious knowledge. Nevertheless, there are also challenges touching on the issue of consciousness: For instance, Maia and McClelland (2004) play down the actual role of the unconscious processing of the somatic markers, claiming that the IGT actually promotes conscious rather than unconscious mentation. As a response, Damásio and colleagues emphasize that the SMH does not disregard the role of consciousness in decision making, seeing unconscious processes as assisting rather than determining it (cf. Bechara, Damásio, Tranel, & Damásio, 2005, p. 159). It would be interesting to ally research on the SMH with more explicitly learning-directed tasks. 

##### Unconscious thought theory

It is not clear the extent to which unconscious thought theory (UTT) is a separate theory, as it shares many of its assumptions with what can be broadly called unconscious knowledge (cf. e.g., Dijksterhuis & Nordgren, 2006), though it does not emphasize the cognitive aspect; that is, it does not go into the details of the unconscious processing of information, namely in decision making, its main focus. Rather, UTT merely tries to account for the existence of what it calls “the” unconscious in the empirically observed fact that people seem to make better decisions when they leave it to “the” unconscious to do the job. Although, unlike SMH, it is not so obviously a cognitive hypothesis, it is of interest to a general theory of unconscious knowledge, which, as seen, must per force include decision making.

The first and obvious problem with this “unconscious thought” is the extent to which it is simply inattention, given that most experiments trying to analyse this process rely heavily on simply distracting the participating subjects, diverting their attention to tasks that demand attention, thus hindering concentration on the task actually being tested (e.g., Dijksterhuis, 2004; Dijksterhuis, Maarten, Nordgren, & van Baaren, 2006). This, the proponents of the theory call “sleep on” the decision, “let the unconscious mull,” and “incubation” (Dijksterhuis, 2004).

The basic assumption – what UTT calls the capacity principle (Dijksterhuis & Nordgren, 2006) – is that the limited capacity of “conscious thought” is not the best resource when making complex decisions,^19^ whereas the virtually unlimited capacity of the unconscious to process information makes it the tool of choice in those cases. Other assumptions of the theory are the bottom-up-versus-top-down principle, according to which the unconscious works bottom-up (whereas conscious thought works top-down); the rule principle, stating that unconscious thought gives rough estimates, as against the rule-like and precise conscious thought; and the convergence-versus-divergence principle, characterizing the unconscious as divergent, thus opposed to the convergence believed to characterize conscious thought (Dijksterhuis & Nordgren, 2006).

**Figure 4.**
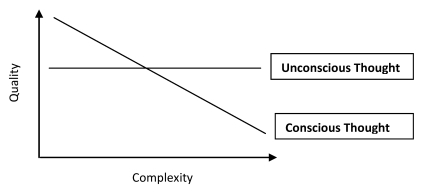
The relation complexity – quality in conscious and unconscious decision making. Adapted from “A Theory of Unconscious Thought” by A. Dijksterhuis and L. F. Nordgren, 2006, *Perspectives on Psychological Science, 1*, p. 103.

The main argument is that whereas complexity greatly interferes with conscious thought, thus often resulting in bad choices, the unconscious is not affected by it (see Figure 4). The assumptions underlying this conclusion are in fact not far from some of those shared by other theories of unconscious knowledge: For instance, as seen above, research in artificial grammars rests in large measure on the assumption that complex grammatical rules are more easily learned unconsciously. However, UTT does not share the fundamental assumptions of the other theories of unconscious cognition: that the stimuli are not consciously perceived (cf. e.g., the double visual stream hypothesis), are not consciously – that is, strategically – learned (cf. the assumptions of research in artificial grammars), or are not consciously accessible (cf. research into implicit memory). UTT simply claims that consciously learned information of a complex kind, but in part or even largely accessible at any time during a decision making task, is better processed when attention is diverted from it.

Thus, it is certainly difficult to see in which way it might contribute to our comprehension of unconscious knowledge, in that the cognitive factors involved are not clear, but in this section, as in most of this text, we are interested in how unconsciously processed knowledge supports successful behaviour. Allowing for the fact that consciously learned and stored information may be processed wholly unconsciously and nevertheless result in optimal, or rational decisions,^20^ we may accept this as a cognitive process with distinctive properties; according to A. Dijksterhuis, this “incubation”^21^ results in “clearer, more polarized, and more integrated representations in memory” (Dijksterhuis, 2004, p. 586; Dijksterhuis et al., 2009). This, the assumption that decision making is better when one lets unconscious processes take care of the job, faces criticism on both the theoretical and methodological levels (e.g., Acker, 2008; González-Vallejo, Lassiter, Bellezza, & Lindberg, 2008).

## Conclusion

Despite much evidence in favour, the claim that there is a specific, qualitatively distinct unconscious kind of knowledge remains controversial. Whether one likes – or admits – it or not, much of the controversy surrounding this field of research aims to discredit it, not so much as being pseudo-scientific, but simply as being methodologically faulty and theoretically wrong. Perhaps, in the name of a sacrosanct rationalism that still equates reason and other “higher” cognitive faculties with consciousness, the objective of many – though obviously not all – critics seems to be the straightforward refutation of the hypothesis that there is unconscious knowledge. The main issue is not prima facie one of demarcation between science and pseudo-science: Experiments are repeated with the objective of refuting positive findings, and criticism targets both the assumptions and the methods of the diverse theories of unconscious perception and cognition. The falsification involved seems to aim at showing that “consciousness does it”, that is, the replacement of theories invoking unconscious processes by a theory of an all-encompassing consciousness; when consciousness does not easily account for the phenomenon, then favourable findings are attributed to methodological weaknesses (e.g., Dulany et al., 1984; Shanks & St. John, 1994). These – in particular the latter – challenges are, however, to be taken seriously. Hence, the need to distinguish clearly between measures of conscious and unconscious perceptual experience. This process led to the advocacy of indirect measures (e.g., Marcel, 1983a) and to the proposal to adopt subjective (vs. objective) measures of awareness (e.g., Merikle, 1992), which rely on what has been dubbed the subjective threshold (the point at which subjects do not know that they know that a stimulus was presented), and also on the ability attributed to humans of having higher order thoughts (being aware of their own mental states; e.g., Dienes, 2007).

If one sides with a definition of knowledge as the establishment of successful relations with the environment (James, 1904), then empirical data in behavioural and (neuro)cognitive psychology suggests strongly that there is a qualitatively distinct kind of knowledge, acquired, stored, and recalled in a wholly unconscious way. Concerning the acquisition of this kind of knowledge, research with artificial grammars and with other paradigms has shown that highly complex systems of rules can be learned and thus correctly applied without improving explicit knowledge of the systems. In a different vein, but with the same objective in view, research into cognition in states of anaesthesia and, to a lesser degree, in coma and in sleep has secured some results that indicate the unconscious processing of material presented in those states in which consciousness is (more) safely ruled out. Studies on implicit memory with unimpaired subjects and subjects with impaired memory have provided evidence that there are specific ways, functionally and anatomically differentiated, of storing and recalling information without awareness. The overall focus of research into unconscious knowledge is the “knowledgeable” behaviour of subjects in the absence of metaknowledge concerning their own epistemic states: Work inspired by the dual stream hypothesis in visual deficits, perhaps better than any other field, shows that individuals can behave successfully by relying only on unconscious mental states, such as unconscious beliefs and intentions. These studies corroborate one of the major tenets of unconscious cognition, to wit, that unconscious knowledge is solely procedural, remaining inaccessible to consciousness and verbalization. Although at first sight not primarily, or at all, concerned with issues of unconscious knowledge, the somatic marker hypothesis and what is known as unconscious thought theory might be seen as contributing to the assumption that one can decide, securing beneficial results, by resorting to unconscious forms of knowledge processing alone. The diverse theories involved share basic assumptions and have many methodological methods in common that call for a unification of the field of unconscious knowledge. This would undoubtedly strengthen the individual theories on this particular subject against the many challenges the hypothesis of an unconscious knowledge still faces contemporarily. Corroborating evidence from emerging and recently developing research in topics such as implicit learning in schizophrenia (e.g., Danion, Meulemans, Kauffmann-Muller, & Vermaat, 2001) and information processing during pre-natal development (e.g., Kisilevsky et al., 2004), while adding to the already staggering complexity of the discussion in relation to consciousness, promises to enrich the field of research in unconscious knowledge.

## Footnotes

1 This cognitive distinction is often paralleled with the epistemological dichotomy between knowledge-that and knowledge-how (e.g., Cohen & Squire, 1980). This is a dichotomy attributed to Ryle (1949), which actually faces much opposition within epistemology (e.g., Stanley & Williamson, 2001), and which is not relevant to the view of knowledge adopted in this study. For instance, speaking a language as a native speaker, and applying this distinction, while being a kind of knowledge-how, it is necessarily a knowledge-that, too (this does not mean that the speaker has to have academic knowledge of their native language; see the case of young fluent speakers). In the case of such a complex competence as speaking a mother language (e.g., individuals with serious cognitive deficits can speak their native languages with remarkable accuracy), it is not at all clear where knowledge-how ends and knowledge-that begins, and vice-versa, which renders the distinction greatly uninformative for our ends. In other words, procedural knowledge is also knowledge of facts and rules, and declarative kinds of knowledge may comprise procedures.

2 This often seems to be the case; for instance, Allen and Reber (1980) showed that two years after a 10-15 min exposure to an artificial grammar, subjects were significantly more likely to assign grammatical status to test items learned in an implicit mode: “While some blurring of structure knowledge comes with time, and subjects report that immediate intuitive apprehension of grammaticality is somewhat harder to come by, knowledge gained in the implicit mode is persistent in both form and quality” (Allen & Reber, 1980, p. 184). Short-lived forms of unconscious knowledge (e.g., motor representations) have been reported, but their short duration might be explained by the interference or contamination by subsequent conscious representations (e.g., Rossetti, 1998). This orchestration with conscious forms of knowledge might also explain instances in which unconscious knowledge appears less inflexible or less rigid (Kiefer, 2007; Kunde, Kiesel, & Hoffmann, 2003; see Feature 4 in this section).

3 That is, they speak the language to which they were exposed correctly, regardless of the correctness of that language in normative terms.

4 Or homologous structures, like the avian Wulst; for this homology and its relation to consciousness, see Butler and Cotterill (2006).

5 Briefly, in his first topographical elaboration of the psychic apparatus (Freud, 1900/1958, 1915/1968), the unconscious is seen as the “place” or “system” where representatives of instinctual needs strive in order to find motor expression (satisfaction) by following somehow predictable processes (the primary process: condensation and displacement). This scenario, ruled by the pleasure principle, is greatly complicated in the second elaboration of the psychic apparatus. Here, beyond the pleasure principle (cf. Freud, 1920/1961), unconscious psychic processes (no longer confined to a “location” but seen as structural: the id can be said to be a deep structure, whereas the ego is a surface structure, in contact with reality; cf. Freud, 1923/1961) are ruled by the compulsion to repeat that can “blindly” seek the very destruction of the organism (the death drive; see Freud, 1920/1961).

6 For instance, recent findings suggest that unconscious processing might actually be subject to some top-down control (e.g., Ansorge & Neumann, 2005; Kiefer, 2007).

7 See Adams (1957) for an exhaustive review of research on “behaviour without awareness” from the late 1800s to the 1950s.

8 See Miller (1939, pp. 562-565) for a brief account of early experimental studies in subliminal perception.

9 Although Fechner already talked of unconscious sensations, his attempt to translate psychological findings and hypotheses into the language of mathematics made this conception rather unintelligible: He actually claimed that below the absolute threshold the subject “perceived less than nothing” (cf. Fechner, 1860).

10 A common bias concerning unconscious processing is that it is reduced to mere automatisms; cases such as blindsight show that this is unlikely to be the case, and that what goes on is actually the formation of unconscious complex, higher mental states, such as beliefs and intentions (e.g., Vakalopoulos, 2005, p. 1185).

11 Other experimental paradigms not directly appealing to the dual visual stream hypothesis that corroborate the hypothesis of an unconscious discrimination are, for instance, the different ways patients scan unfamiliar and familiar faces (Rizzo, Hurtig, & Damásio, 1987) and their shorter reaction times in matching familiar faces (de Haan, Young, & Newcombe, 1987).

12 This is an important specification, as the umbrella terms *hemiagnosia, hemineglect, hemispatial neglect*, etc. include dysfunctions in other sensory modalities.

13 In a “classical” auditory version (e.g., MacKay, 1973), at the same time that the subject shadows (attends to and repeats aloud) a sentence like *They stood by the bank* presented to one ear, the word *water* (or *money*) is presented to the other ear.

14 Also of import was the conclusion suggested by the results that Stroop-type effects do not occur only at the stage of response.

15 For example, as managers of a sugar production factory, reaching and maintaining a specified level of sugar output, by varying the number of working employees.

16 There are three main theoretical accounts of memory: the already known multiple memory system, and the activation and processing accounts (e.g., Schacter, 1987, p. 511).

17 Well illustrated by states of somnambulism, commonly occurring during a deep sleep state (Stages 3 and 4) in which subjects answer appro-priately to sensory input (e.g., Jacobson, Kales, Lehmann, & Zweizig, 1965).

18 This against the claims of many Soviet and East European studies in the 1960s; however, it might be the case that their results were due to different methodological approaches, namely the fact that the material to be learned was presented before sleep onset (cf. Aarons, 1976).

19 These being decisions with multiple factors to them.

20 This is one factor that greatly weakens UTT, as the question of normativity and rationality is far from uncontroversial (e.g., Shafir & LeBoeuf, 2002).

21 It is important to remark that the theoretically vague “incubation” is not usually seen as involving unconscious thought processes (e.g., Seabrook & Dienes, 2003).
